# Pneumomediastinum Masquerading As Superior Vena Cava Obstruction in a Patient With Metastatic Colorectal Cancer: A Diagnostic Challenge

**DOI:** 10.7759/cureus.96301

**Published:** 2025-11-07

**Authors:** Ariba Ali, Ishaque Rafai, Sana Javed Malik

**Affiliations:** 1 Oncology, Queen Alexandra Hospital, Portsmouth, GBR; 2 Internal Medicine, Queen Alexandra Hospital, Portsmouth, GBR; 3 Acute Medicine, Queen Alexandra Hospital, Portsmouth, GBR

**Keywords:** chemotherapy, colorectal cancer, macklin effect, spontaneous pneumomediastinum (spm), superior vena cava obstruction (svco)

## Abstract

Pneumomediastinum refers to the presence of free air within the mediastinum and is most commonly associated with trauma, instrumentation, or barotrauma, but may rarely occur spontaneously. In patients with thoracic malignancy, pneumomediastinum can mimic other oncologic emergencies such as superior vena cava obstruction (SVCO), creating diagnostic uncertainty.

We present the case of a 57-year-old woman with metastatic recurrent colorectal carcinoma receiving palliative FOLFIRI (folinic acid, fluorouracil, and irinotecan) and panitumumab who was admitted with recurrent respiratory infections. During a subsequent hospitalization, she developed acute cervicofacial swelling with dysphagia and visible collateral chest wall veins, prompting empirical high-dose steroids and anticoagulation for suspected SVCO. Contrast-enhanced CT performed the same day demonstrated extensive pneumomediastinum with cervicofacial subcutaneous emphysema, but no venous obstruction or tumor progression. The leading etiologies considered included barotrauma and the Macklin effect associated with cough and asthma exacerbation; iatrogenic causes such as port-a-cath leak were excluded. She improved with conservative management (oxygen, bronchodilators, steroid taper, and antibiotics as indicated) and was discharged. Due to persistent hilar disease and recurrent respiratory compromise, the thoracic multidisciplinary team later proposed palliative surgical debulking.

This case highlights the importance of prompt imaging and multidisciplinary assessment in differentiating pneumomediastinum from SVCO to avoid unnecessary invasive interventions.

## Introduction

Colorectal carcinoma is among the most common malignancies worldwide and frequently metastasizes to the lungs, particularly in advanced stages of disease [1]. Pulmonary metastases may arise synchronously or metachronously following resection of the primary tumor, often presenting with cough, dyspnea, or recurrent respiratory infections. In such patients, the differential diagnosis of respiratory symptoms is broad, ranging from infection and treatment-related toxicity to malignant progression or therapy-related complications.

When an oncology patient presents with acute cervicofacial swelling, particularly in the context of hilar recurrence, clinicians often suspect superior vena cava obstruction (SVCO) - a recognized oncologic emergency that necessitates prompt identification and management [2]. SVCO typically results from tumor compression of the superior vena cava, leading to impaired venous return and the classic presentation of facial and upper limb edema, venous distension, and dyspnea.

However, pneumomediastinum - the presence of free air within the mediastinum - is an uncommon but important mimic of SVCO. It may occur spontaneously through the Macklin effect, in which alveolar rupture allows interstitial air to dissect along bronchovascular sheaths into the mediastinum [3,4]. Alternatively, it can result from infection, asthma exacerbation, barotrauma, or iatrogenic instrumentation, including thoracic procedures or central line insertions [5,6]. The clinical overlap between SVCO and pneumomediastinum poses a diagnostic challenge, especially in oncology patients, where both disease progression and treatment complications are plausible.

In such cases, contrast-enhanced imaging plays a pivotal role in differentiating these conditions, as prompt and accurate diagnosis directly impacts management. Misinterpretation can lead to unnecessary anticoagulation, corticosteroid therapy, or invasive procedures.

We report the case of a 57-year-old woman with metastatic colorectal carcinoma who developed acute cervicofacial swelling initially suspected to represent SVCO but was ultimately diagnosed with extensive pneumomediastinum. This case underscores the importance of maintaining a broad differential diagnosis, careful review of imaging, and multidisciplinary collaboration in managing complex oncology presentations.

## Case presentation

A 57-year-old woman with metastatic recurrent colorectal carcinoma involving the right middle lobe and hilar lymph nodes was undergoing palliative chemotherapy with FOLFIRI (folinic acid, fluorouracil, and irinotecan) in combination with panitumumab.

Her oncological history began in 2019, when she underwent a robotic anterior resection for rectal adenocarcinoma (pT3N2, MMR proficient, RAS wild-type), followed by adjuvant oxaliplatin-capecitabine chemotherapy. In 2022, she developed pulmonary metastases and subsequently underwent a right lower lobe segmentectomy, during which an incidental carcinoid tumour was also resected. Between 2023 and 2025, she experienced multiple episodes of pulmonary recurrence, managed sequentially with oxaliplatin-capecitabine and later panitumumab combined with FOLFIRI, resulting in alternating periods of partial remission and mild progression.

**Figure 1:**
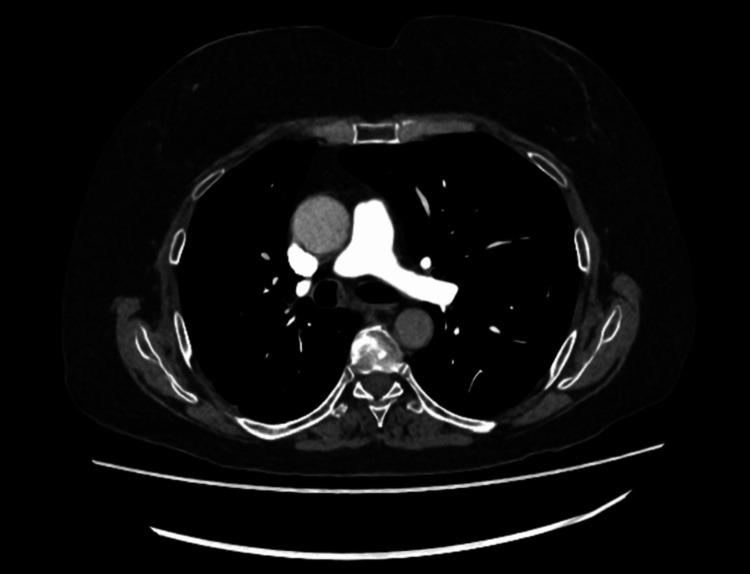
CT pulmonary angiogram demonstrating no pulmonary embolism. No new nodules or effusions were observed. This imaging confirmed disease stability and excluded thromboembolic pathology as the cause of the patient’s respiratory symptoms.

In early September 2025, she presented with a short history of increasing cough and breathlessness. A CT pulmonary angiogram (CTPA) ruled out pulmonary embolism but demonstrated a stable right hilar mass (Figure 1). She was treated for an asthma exacerbation with oral doxycycline for five days, along with nebulised bronchodilators, and improved sufficiently to be discharged.

Two weeks later, she was re-admitted with a persistent cough and progressive dyspnoea. On admission, she was afebrile, with oxygen saturations of 97% on room air, a respiratory rate of 18 breaths/min, a heart rate of 100 bpm, and a blood pressure of 130/80 mmHg. Laboratory investigations revealed a white cell count of 12.1 × 10^9^/L and C-reactive protein of 65 mg/L. Renal and hepatic function remained within normal limits. Sputum culture grew *Klebsiella pneumoniae*, sensitive to piperacillin-tazobactam, for which intravenous antibiotics were commenced.

On the third day of admission, she developed acute right-sided facial and neck swelling associated with erythema and a sensation of tightness. An allergic reaction was initially suspected, and she received antihistamines with minimal improvement. Within 24 hours, the swelling worsened and was accompanied by dysphagia, visible collateral veins, and palpable crepitus along the neck and upper chest. SVCO was suspected, and high-dose intravenous dexamethasone (8 mg twice daily) and prophylactic anticoagulation were initiated.

**Figure 2:**
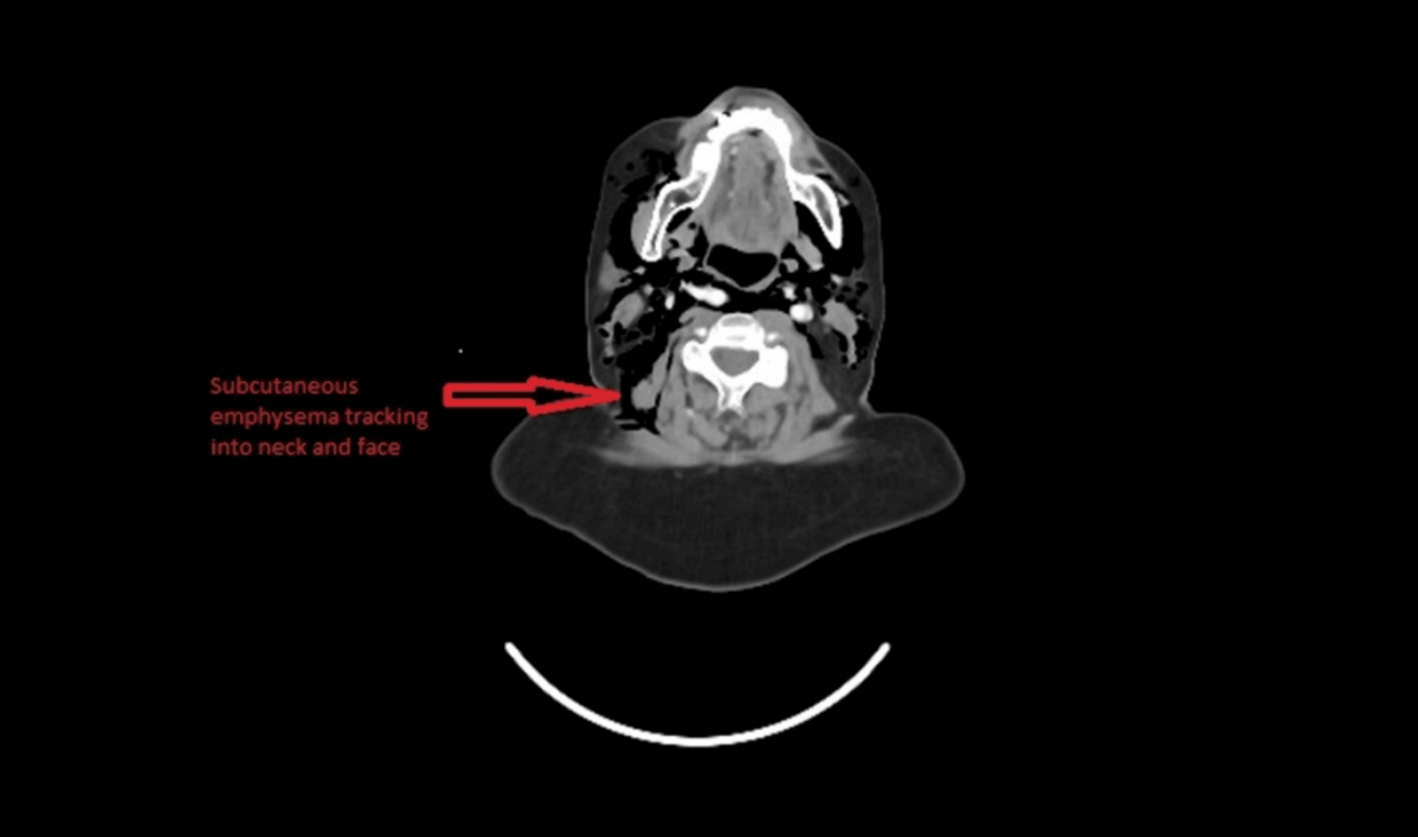
CT neck and chest on 23/09/25. Contrast-enhanced CT of the chest and neck showing subcutaneous emphysema tracking into the neck and face. There is no evidence of superior vena cava obstruction, mass expansion, or port-a-cath leakage, confirming a non-compressive etiology for the swelling.

A contrast-enhanced CT scan of the chest and neck (Figure 2) revealed extensive pneumomediastinum and cervicofacial subcutaneous emphysema, with no evidence of SVCO or tumour progression. The previously inserted port-a-cath, placed one month earlier, appeared intact without leakage. Following review by the cardiothoracic and oncology multidisciplinary teams, the pneumomediastinum was considered most likely spontaneous, secondary to the Macklin effect triggered by coughing or airway inflammation.

**Figure 3:**
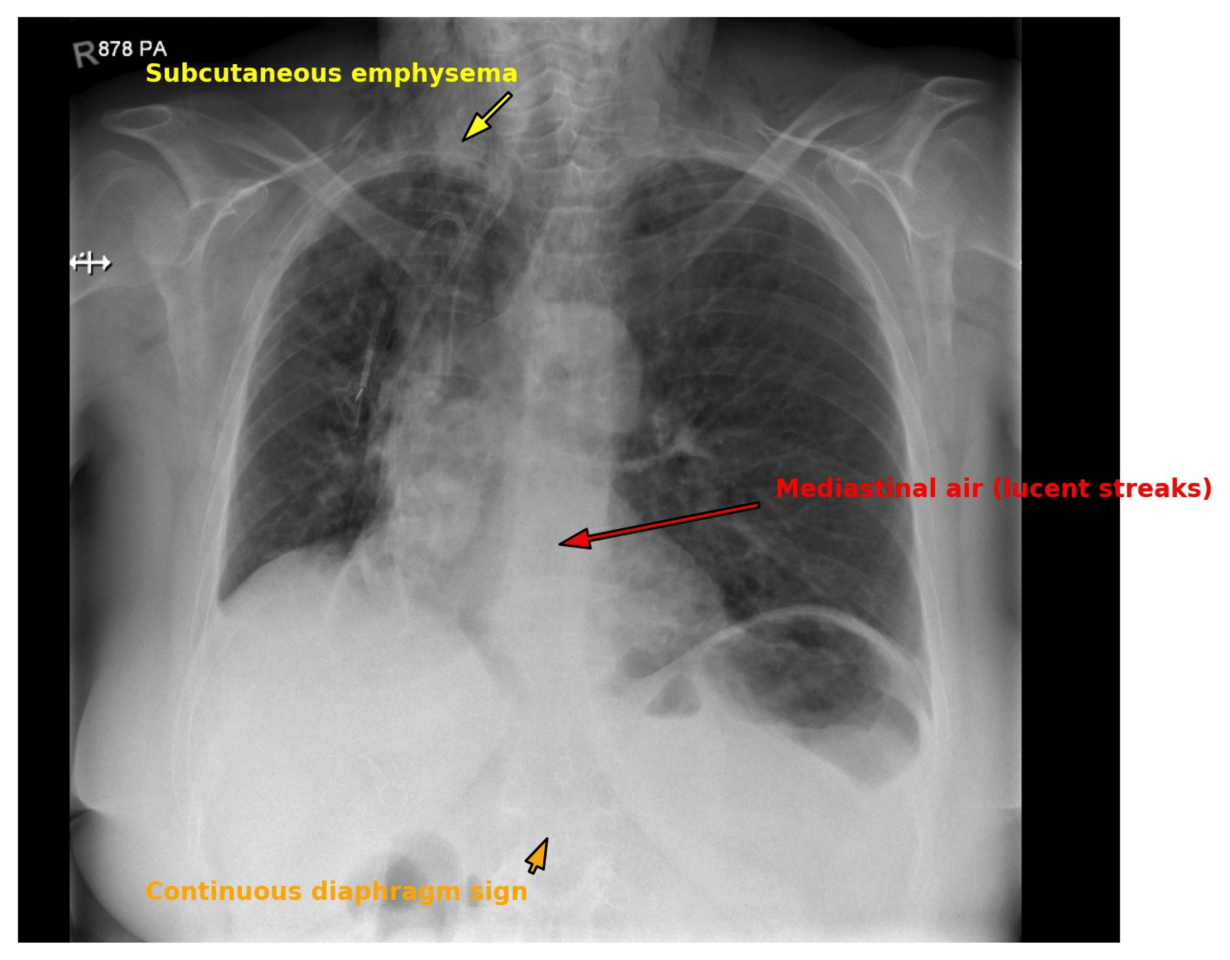
Chest X-ray. Posteroanterior chest X-ray showing pneumomediastinum. Subcutaneous emphysema is seen in the upper chest and neck (yellow arrow). Mediastinal air outlines mediastinal structures (red arrow), with a continuous diaphragm sign (orange arrow) beneath the heart. No pneumothorax is observed.

She was managed conservatively with corticosteroids, bronchodilators, supplemental oxygen, and close monitoring. Over the next three days, her facial swelling, crepitus, and respiratory symptoms gradually resolved. A follow-up chest X-ray (Figure 3) demonstrated improving subcutaneous emphysema and no pneumothorax.

After clinical stabilization, she was discharged with a tapering course of oral steroids and scheduled follow-up with oncology and cardiothoracic surgery. Given the persistent hilar mass and recurrent respiratory symptoms, the cardiothoracic multidisciplinary team later proposed palliative debulking surgery of the lung mass to relieve obstruction and improve symptom control.

## Discussion

Spontaneous pneumomediastinum (SPM) is a rare clinical entity characterized by free air within the mediastinum without an overt traumatic, iatrogenic, or perforation-related cause. Most often, it follows a benign, self-limited course, but it can mimic critical thoracic emergencies such as SVCO or even anaphylaxis. The most accepted pathophysiological mechanism is the Macklin effect, in which alveolar rupture allows air to dissect along bronchovascular sheaths into the mediastinum after an acute increase in intra-alveolar pressure, such as during coughing, vomiting, or asthma exacerbation [3,4].

In oncology patients, pneumomediastinum is uncommon but may arise secondary to tumor necrosis, chemotherapy-induced alveolar injury, infection, or mechanical ventilation [5,7]. In rare instances, prior lung surgery or radiotherapy may weaken pulmonary structures, increasing susceptibility [8]. In our patient, recurrent infection, severe coughing, and underlying pulmonary metastases likely precipitated the event via the Macklin mechanism.

Clinically, the presentation with cervicofacial swelling, collateral vein prominence, and dyspnea closely resembled SVCO, a known complication of mediastinal malignancy [2]. However, the presence of palpable crepitus on physical examination, along with imaging showing no venous obstruction, pointed toward pneumomediastinum instead. Contrast-enhanced CT is the gold standard for distinguishing these conditions by demonstrating air tracking and excluding other causes such as esophageal perforation or catheter leak [9].

Most cases of SPM are managed conservatively with rest, supplemental oxygen to facilitate nitrogen washout, analgesia, and close observation, and most resolve within days [10]. In contrast, true SVCO may require corticosteroids, anticoagulation, radiotherapy, or endovascular stenting depending on the severity and etiology [11]. Misdiagnosing SPM as SVCO may lead to unnecessary and potentially harmful interventions.

In this patient, CT imaging confirmed extensive pneumomediastinum without vascular compression. Conservative management led to complete resolution. Given her persistent hilar disease and recurrent respiratory compromise, the thoracic team later proposed palliative debulking to reduce the risk of further airway compromise.

This case underscores the importance of maintaining a broad differential diagnosis in oncology patients presenting with cervicofacial swelling. Subtle findings, such as crepitus, may guide correct diagnosis. Prompt imaging, expert interpretation, and multidisciplinary collaboration between oncology, radiology, pulmonary, and thoracic surgery teams are crucial for optimized care.

## Conclusions

Acute cervicofacial swelling in patients with hilar metastases often raises concern for SVCO, a recognized oncologic emergency. This case demonstrates that pneumomediastinum, although rare, can closely mimic SVCO, potentially leading to misdiagnosis and unnecessary interventions. Clinicians should maintain a high index of suspicion, particularly in patients with predisposing factors such as asthma exacerbation, coughing, or recent respiratory infection. Careful evaluation with contrast-enhanced imaging, recognition of subtle signs such as palpable crepitus, and a multidisciplinary approach are essential to establish the correct diagnosis. Most cases can be managed conservatively; however, in select patients with persistent obstruction or symptoms, surgical interventions such as debulking may be considered for palliation and symptom relief. This report underscores the importance of integrating clinical, radiological, and multidisciplinary insights to optimize patient care in complex oncology scenarios.
